# Aptamer-based self-assembled nanomicelle enables efficient and targeted drug delivery

**DOI:** 10.1186/s12951-023-02164-y

**Published:** 2023-11-09

**Authors:** Ganghui Chen, Dongsheng Mao, Xuan Wang, Jingqi Chen, Chao Gu, Shuqin Huang, Yu Yang, Fang Zhang, Weihong Tan

**Affiliations:** 1 Institute of Molecular Medicine (IMM), Shanghai Jiao Tong University School of Medicine, Renji Hospital, College of Chemistry and Chemical Engineering, Shanghai Jiao Tong University, Shanghai, 200240 China; 2 College of Biological Science and Engineering, Fuzhou University, Fuzhou, 350108 People’s Republic of China

**Keywords:** Aptamer micelles, Self-assembly, Specific cell recognition

## Abstract

Nucleic acid aptamer-based nanomicelles have great potential for nanomedicine and nanotechnology applications. However, amphiphilic aptamer micelles are known to be inherently unstable upon interaction with cell membranes in the physiological environment, thus potentially compromising their specific targeting against cancer cells. This flaw is addressed in the present work which reports a superstable micellar nanodelivery system as an amphiphilic copolymer self-assembled micelle composed of nucleic acid aptamer and polyvalent hydrophobic poly(maleic anhydride-alt-1-octadecene) (C_18_PMH). Using Ce6 as a drug model, these C_18_-aptamer micelles exhibit efficient tumor-targeting and -binding ability, facilitating the entry of Ce6 into targeted cells for photodynamic therapy. In addition, they can be loaded with other hydrophobic drugs and still demonstrate favorable therapeutic effects. As such, these C_18_-aptamer micelles can serve as a universal platform for loading multiple drugs, providing a safer and more effective solution for treating cancer.

**Supplementary Information:**

The online version contains supplementary material available at 10.1186/s12951-023-02164-y.

## Introduction

Aptamers, single-stranded DNA or RNA molecules, are capable of adopting specific secondary and tertiary structures, allowing them to selectively bind to target molecules [1, 2], including metal ions [3], metabolites [4, 5], and proteins [6, 7]. Compared with biological molecular ligands, such as antibodies, peptides or small molecules [8, 9], aptamers exhibit fast and reliable synthesis, convenient modification, high chemical stability, and low immunogenicity [10]. Moreover, because aptamers can readily change conformation, while exhibiting these merits, a broad range of aptamer-based nanomaterials can be constructed with controlled size, structure, shape, and function [11–13]. For this reason, aptamer-based biomaterials have received considerable attention [14], including, for example, functional DNA nanostructures [15, 16], DNA micellar polymers [17] and DNA hydrogels [18, 19]. However, some nanosystems built from aptamer-based nanomaterials may, nonetheless, show unstable interactions with protein or cell membrane, leading to unpredictable aptamer leakage and nonspecific targeting [20].

Amphiphilic DNA nanomaterials, a common type of aptamer-based biomaterials, are created by combining hydrophobic polymer molecules with hydrophilic aptamers. These nanomaterials can spontaneously assemble into spherical DNA micelles in aqueous environments [21, 22]. Studies have demonstrated the feasibility of using nucleic acid aptamer micelles to specifically target cancer cells [23]. However, the plasma membrane of cells is primarily composed of a lipophilic phospholipid bilayer, which poses a challenge to maintaining the stability of these micelles in the physiological environment [24]. To explain, hydrophobic components of the micelle structure can easily insert into, or penetrate, the membrane, causing the micelle to disassemble into monomers, thereby losing its recognition ability and, ultimately, undermining targeted drug delivery [25]. Numerous approaches have been developed to increase the stability of micelle structures, particularly in the context of DNA micelles. Two notable strategies for stabilizing these micelles involve crosslinking, which has been explored by both the Mirkin group and the Tan group [23, 26]. However, crosslinking aptamer micelles in these approaches requires additional steps, thus limiting their broader applicability. Another approach uses stable polyvalent hydrophobic chains further modified with nucleic acid aptamers. This strategy can improve micelle stability, as well as targeting affinity, and offer promising avenues for future research.

**Scheme 1:**
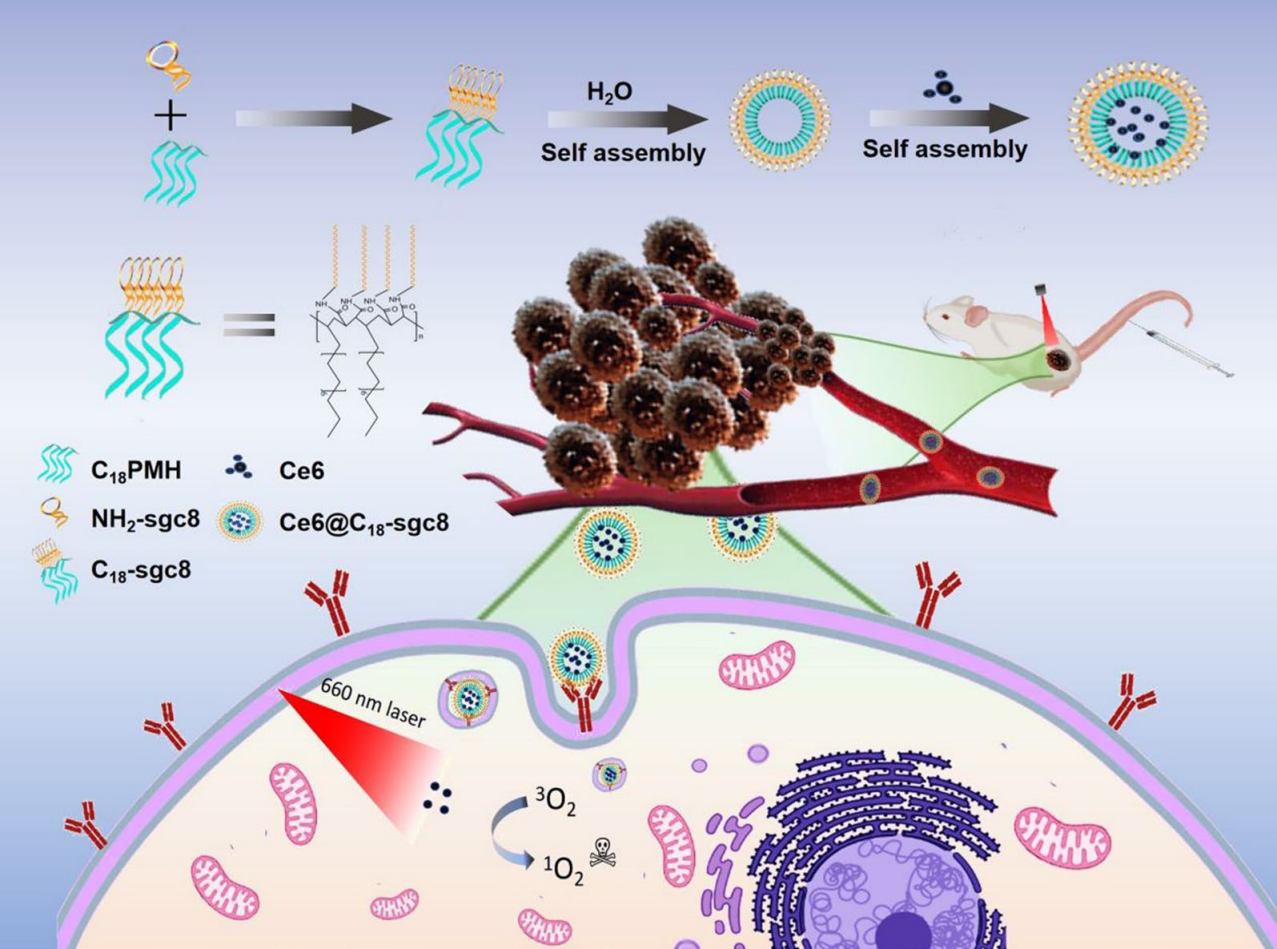
Schematic illustration of the synthesis method of C_18_-sgc8 and its application for targeted photodynamic therapy

The present paper aimed to develop a stable DNA-lipid micelle that does not require crosslinking aptamers or the incorporation of stable polyvalent hydrophobic chains modified with nucleic acid aptamers (Scheme 1). Instead, polyvalent hydrophobic poly(maleic anhydride-alt-1-octadecene) (C_18_PMH) is used as the stable polyvalent hydrophobic end and DNA as the hydrophilic end. This is achieved by reacting C_18_PMH, which contains a large number of C18 chains and anhydrides [27], with a large number of NH2-aptamers to form an amphiphilic polymer with a large number of C18 and DNA chains in tandem. Unlike conventional DNA micelles, the C_18_-aptamer does not require further crosslinking and remains stable in the cellular environment. Its multivalent nature and stable structure also provide excellent binding specificity to target cells. To verify that C_18_-aptamer has stable targeting, we selected aptamer sgc8, which has an affinity for PTK7 on the cell membrane, and performed photodynamic therapy by loading the photosensitizer Ce6 in the C_18_-aptamer micelles. The resulting Ce6@C_18_-sgc8 complex showed excellent performance in targeted photodynamic therapy (PDT) at the cellular level, facilitating Ce6 cell internalization and normalizing singlet oxygen generation (SOG) under laser irradiation, ultimately leading to apoptosis. This C_18_-aptamer micelle also effectively targets and kills tumor cells loaded with other hydrophobic drugs. The IC_50_ of C_18_-sgc8 DNA lipid micelles is reduced by 71.54%, 60.69% and 56.56% compared to that of free Doxorubicin (DOX), paclitaxel (PTX) and Ce6, respectively. These results demonstrate that the C_18_PMH can be used as a universal aptamer platform for targeted delivery of multiple drugs and that it is a promising therapeutic strategy for tumor treatment.

## Results and discussion

**Fig. 1:**
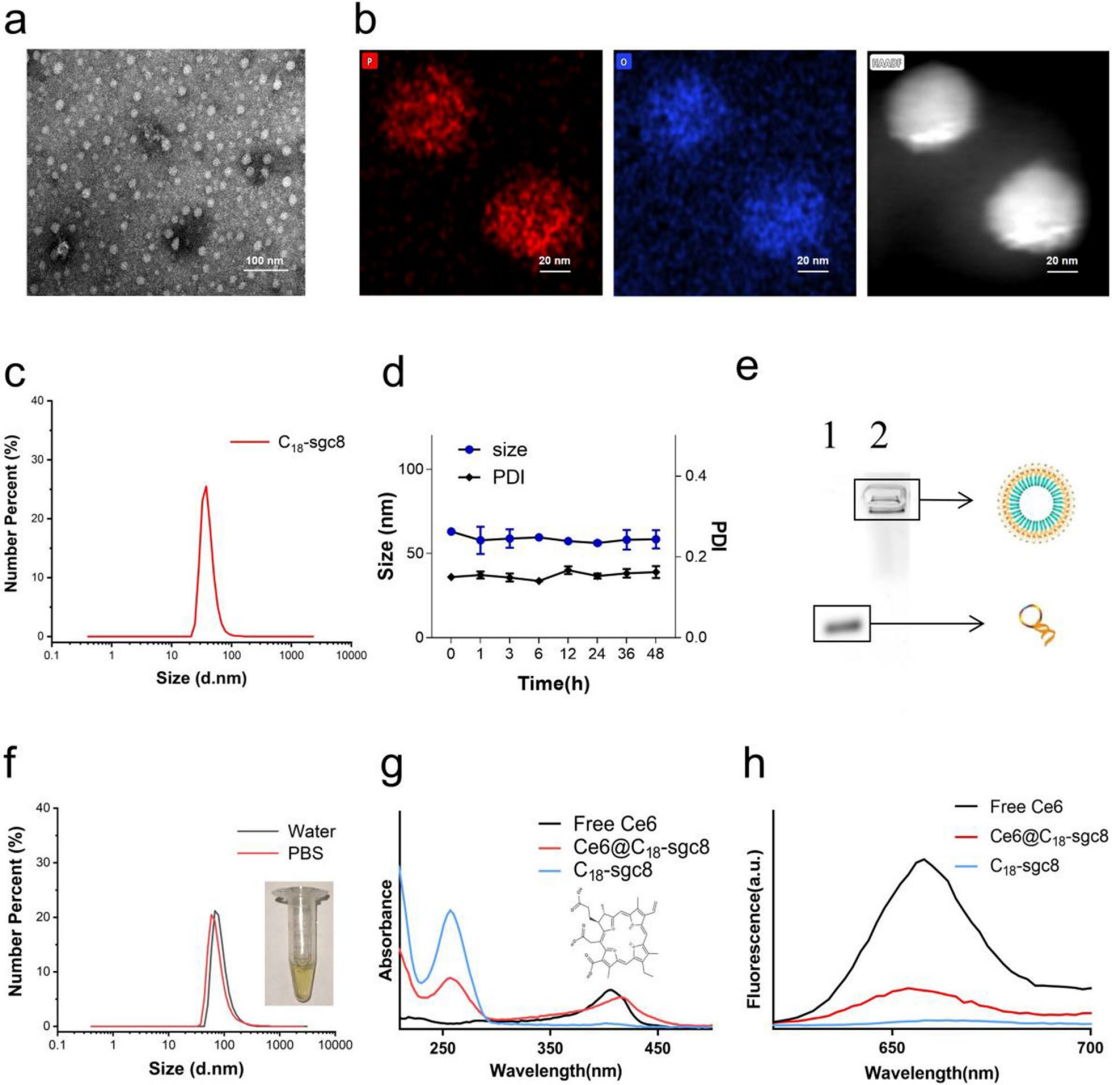
Characterization of C_18_-sgc8 and Ce6@C_18_-sgc8. **a** TEM images of the self-assembly of C_18_-sgc8 in water to form nanomicelles. **b** TEM mapping images of C_18_-sgc8. **c** DLS measurements of C_18_-sgc8 showing a median diameter of 38.8 nm. **d** DLS and PDI of C_18_-sgc8 nanometer micelles in water at 48 h. **e** Verification of the coupling of C_18_PMH and NH_2_-aptamers through 4% agarose gel electrophoresis. A buffer solution containing GelRed dye and NH_2_-sgc8 or C_18_-sgc8 was selected, followed by adding to the sample loading hole. After electrophoresis, agarose gel was used to select the GelRed channel in the imaging system for imaging: Lanes 1, sgc8 and 2, C_18_-sgc8. **f** DLS measurements of Ce6@C_18_-sgc8 in water (black) and PBS (red) micelles. **g** UV*–*vis spectra of Ce6, C_18_-sgc8 and Ce6@C_18_-sgc8. **h** Fluorescence spectra of Ce6, C_18_-sgc8 and Ce6@C_18_-sgc8

This study presents a new approach to hydrophobic drug delivery using self-assembling nucleic acid aptamer nanomicelles. C_18_-sgc8 aptamer monomers were synthesized by conjugating the maleic anhydride group in the backbone of poly(maleic anhydride-alt-1-octadecene) (C_18_PMH) with the amino group on NH2-sgc8. The resulting C_18_-sgc8 nanomicelles were then purified by dialysis. Negative staining transmission electron microscopy (TEM) and dynamic light scattering (DLS) analyses revealed that the nanomicelles were spherical in shape with a uniform size distribution (Fig. 1a) and an average particle size of 37.84 nm (Fig. 1c). In addition, TEM-energy dispersive spectroscopy (EDS) mapping revealed a high concentration of P-element within the C_18_-sgc8 nanomicelle (Fig. 1b), confirming that C_18_PMH can successfully modify numerous DNA strands. Furthermore, C_18_-sgc8 nanomicelles were observed to maintain a uniform state for up to 48 h with a poly dispersity index (PDI) below 0.2 and remained stable for a longer period of time (Fig. 1d and Additional file 1: Fig. S1), indicating their potential as a robust drug delivery system. C_18_-sgc8 is a polymer containing a large number of C18 and DNA strands linked in tandem to form stable micelle particles. In addition, as shown in Fig. 1e and 4% agarose electrophoresis confirmed the molecular weight of C_18_-sgc8 nanomicelles to be significantly higher than that of free sgc8. The critical micelle concentration (CMC) value was determined to be approximately 0.01143 mg/mL (Additional file 1: Fig. S2), demonstrating the successful preparation of DNA-based nanomicelles. Taken together, these results point to the promise of this nucleic acid aptamer nanomicelle delivery platform for the efficient and effective delivery of hydrophobic drugs.

Next, C_18_-sgc8 was used as a vehicle to load the hydrophobic photosensitizer Ce6 via hydrophobic-hydrophobic interaction [28], resulting in the formation of drug micelles, hereinafter termed as Ce6@C_18_-sgc8. Notably, loading with Ce6 induced a significant change in the size distribution of the resulting nanomicelles as the median particle size increased from 37.84 to 68.06 nm in water (Fig. 1f). The UV*–*vis spectrum of Ce6@C_18_-sgc8 showed a characteristic peak of DNA at 260 nm, along with a red-shifted peak of loaded Ce6 at 416 nm compared to free Ce6 (Fig. 1g). Fluorescence spectrum analysis further confirmed the successful formation of Ce6@C_18_-sgc8 with a typical emission peak at 660 nm (Fig. 1h). Collectively, these results demonstrate the successful formation of Ce6@C_18_-sgc8 and highlight its potential for drug delivery.

**Fig. 2:**
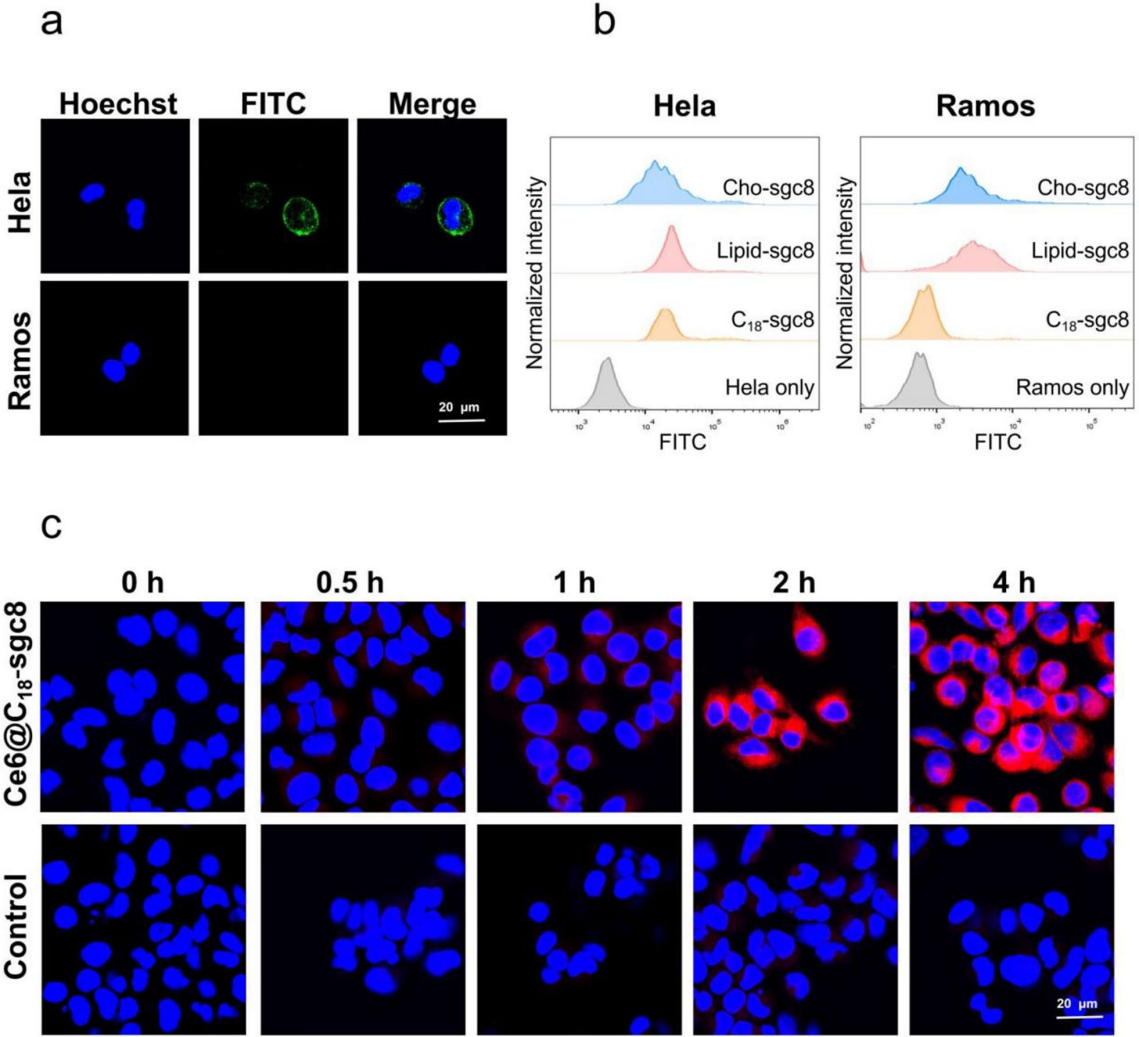
**a** Confocal images of HeLa or Ramos cells incubated with C_18_-sgc8-FITC incubated at 4 °Cfor 1 h, followed by observing the fluorescence of Hoechst and FITC under a confocal microscope. **b** Flow cytometry histogram of HeLa or Ramos cells incubated with C_18_-sgc8-FITC, lipid-sgc8-FITC, or Cho-sgc8-FITC at 4 °C for 1 h. **c** Confocal images of HeLa cells incubated with Ce6@C_18_-sgc8 or free Ce6 at 37 °C for various times as indicated

The targeting ability of C_18_-sgc8 nanomicelles was also studied in comparison to other DNA nanomaterials, such as lipid DNA or cholesterol DNA. The latter nanomaterials have been reported to dynamically disassemble and insert into random cell membranes [29–31], resulting in nonspecific binding to most cells. As noted above, aptamer sgc8 was chosen as the DNA fragment by its specific recognition of and binding to the highly expressed membrane protein PTK7 on HeLa cells, but not Ramos cells [32, 33]. After incubating HeLa cells and Ramos cells with C_18_-sgc8 nanomicelles and imaging with the confocal microscope (Fig. 2a), it can be observed that C_18_-sgc8 micelles selectively bound to HeLa cells, but not Ramos cells, under the same conditions.

To further confirm this selective binding, we incubated Lipid-sgc8, cholesterol-sgc8, and C_18_-sgc8 with HeLa cells or Ramos cells, respectively, for 1 h at 4 °C and then assayed their binding by flow cytometry. Flow cytometry results show that C_18_-scg8 bound only to HeLa cells, whereas lipid-sgc8 and cholesterol-sgc8 bound to both types of cells (Fig. 2b). This demonstrates that C_18_-sgc8 micelles have better specific binding capacity than either lipid-sgc8 or cholesterol-sgc8. This could be attributed to the unique structure of C_18_-sgc8, which is a polymer with a large number of C18 and DNA strands in tandem. This structure facilitates the formation of a more stable micelle structure that prevents the disintegration into individual lipid units when interacting with the cell membrane.

Next, Ce6 was loaded with C_18_-sgc8 micelles to investigate the effect of endocytosis on the intracellular behavior of Ce6@C_18_-sgc8 nanomicelles. HeLa cells were incubated with Ce6@C_18_-sgc8 nanomicelles and free Ce6 for different times and imaged with confocal microscopy (Fig. 2c). Ce6@C_18_-sgc8 quickly endocytosed and showed fluorescence signals of Ce6 in the experimental group, while free Ce6 had difficulty entering HeLa cells. This indicates that C_18_-sgc8 micelles can effectively facilitate the entry of Ce6 into cells.

**Fig. 3:**
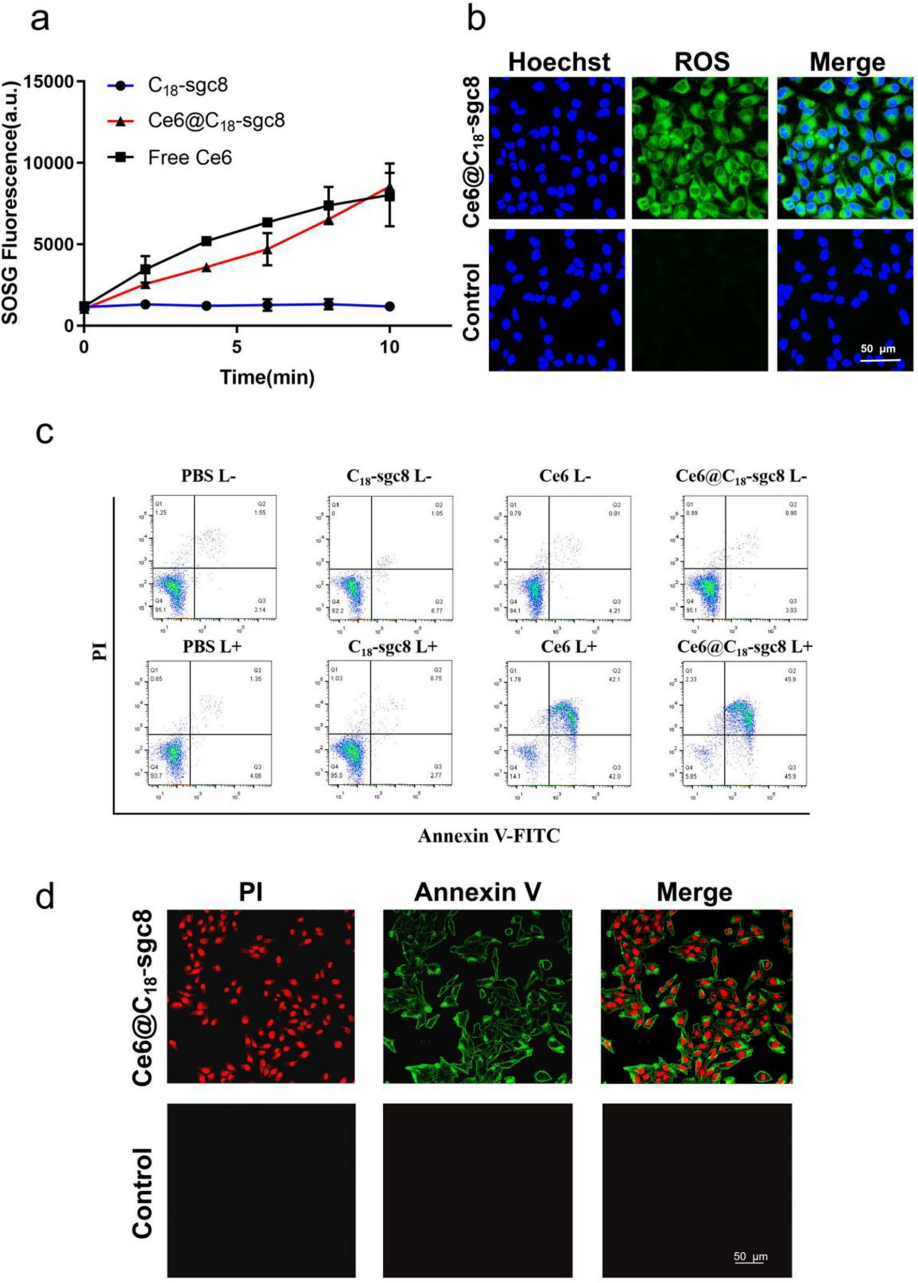
**a** SOSG generation of Ce6@C_18_-sgc8, Ce6, C_18_-sgc8 after 660 nm NIR laser irradiation. **b** Fluorescence intensities of ROS in cells after incubation of Ce6@C_18_-sgc8 under 660 nm NIR laser irradiation. **c** Annexin V-FITC/PI analysis of HeLa cells after incubation with PBS, C_18_-sgc8, Ce6, or Ce6@C_18_-sgc8 under 660 nm NIR laser irradiation. **d** Confocal images of HeLa cells with Annexin V-FITC/PI staining after incubation of Ce6@C_18_-sgc8 under 660 nm NIR laser irradiation

PDT combines light energy with a drug designed to destroy cancerous and precancerous cells after light activation [34]. More specifically, during PDT, a photosensitizer is exposed to specific light wavelengths, leading to the conversion of oxygen in solution into highly reactive singlet oxygen. This change causes cytotoxicity and cell damage or apoptosis [35]. Singlet Oxygen Sensor Green (SOSG) is a single linear oxygen fluorescent probe that is highly selective for ^1^O_2_, and binding to ^1^O_2_ produces a fluorescence signal that can be measured by fluorescence intensity [36, 37]. To investigate whether loading of Ce6 into C_18_-sgc8 affects its ability to produce singlet oxygen, we used SOSG as a probe to detect the level of ^1^O_2_ production. As shown in Fig. 3a, the fluorescence of SOSG does not change significantly in the absence of Ce6 under laser radiation irradiation. However, the fluorescence of free Ce6 and Ce6@C_18_-sgc8Ce6 increases with time, indicating that Ce6 has good photodynamic properties, both before and after assembly. Ce6@C_18_-sgc8 was further explored for its ability to generate singlet oxygen (^1^O_2_) normally in photodynamic therapy at the cellular level. DCFH-DA (2′,7′-dichlorofluorescein diacetate) is often used as a cellular ^1^O_2_ indicator to study the cellular production of ^1^O_2_ in HeLa cells in vitro [38]. As shown in Fig. 3b, after light radiation, significant fluorescence was produced in Ce6@C_18_-sgc8-treated cells as photographed by confocal microscopy, whereas control free Ce6 produced no significant fluorescence signal. This suggests that C_18_-sgc8 can help Ce6 to enter the cells and perform photodynamic therapy, which could be attributed to the binding of sgc8 to overexpressed PTK7, which in turn promotes drug uptake by tumor cells.

PDT is a well-established technique that induces apoptosis through the production of highly reactive singlet oxygen species that can cause cell damage and death [39, 40]. During apoptosis, phosphatidylserine is translocated from the inner to the outer side of the cell membrane. Annexin V is a protein that specifically binds to phosphatidylserine, making it a useful tool for detecting apoptosis [41, 42]. To investigate the apoptotic effect of Ce6@C_18_-sgc8, the Annexin V-FITC/PI kit was used to detect apoptosis by flow cytometric analysis. As shown in Fig. 3c, the fluorescence signal of FITC and PI showed that both free Ce6 and Ce6@C_18_-sgc8 induced massive apoptosis (over 90%) in HeLa cells under light irradiation, suggesting that Ce6@C_18_-sgc8 has good antitumor potential. In addition, Annexin V-FITC/PI staining was observed by confocal microscopy. As shown in Fig. 3d, a large amount of green fluorescence appeared on the membranes of most HeLa cells incubated with Ce6@C_18_-sgc8 after laser irradiation, which resulted from the binding of fluorescein Annexin V to phosphatidylserine on the outside of the apoptotic cell membrane. These results confirm that Ce6@C_18_-sgc8 generates singlet oxygen that linearly depends on light irradiation in a manner that induces apoptosis. This highlights the potential of this nucleic acid aptamer platform for photodynamic therapy.

**Fig. 4:**
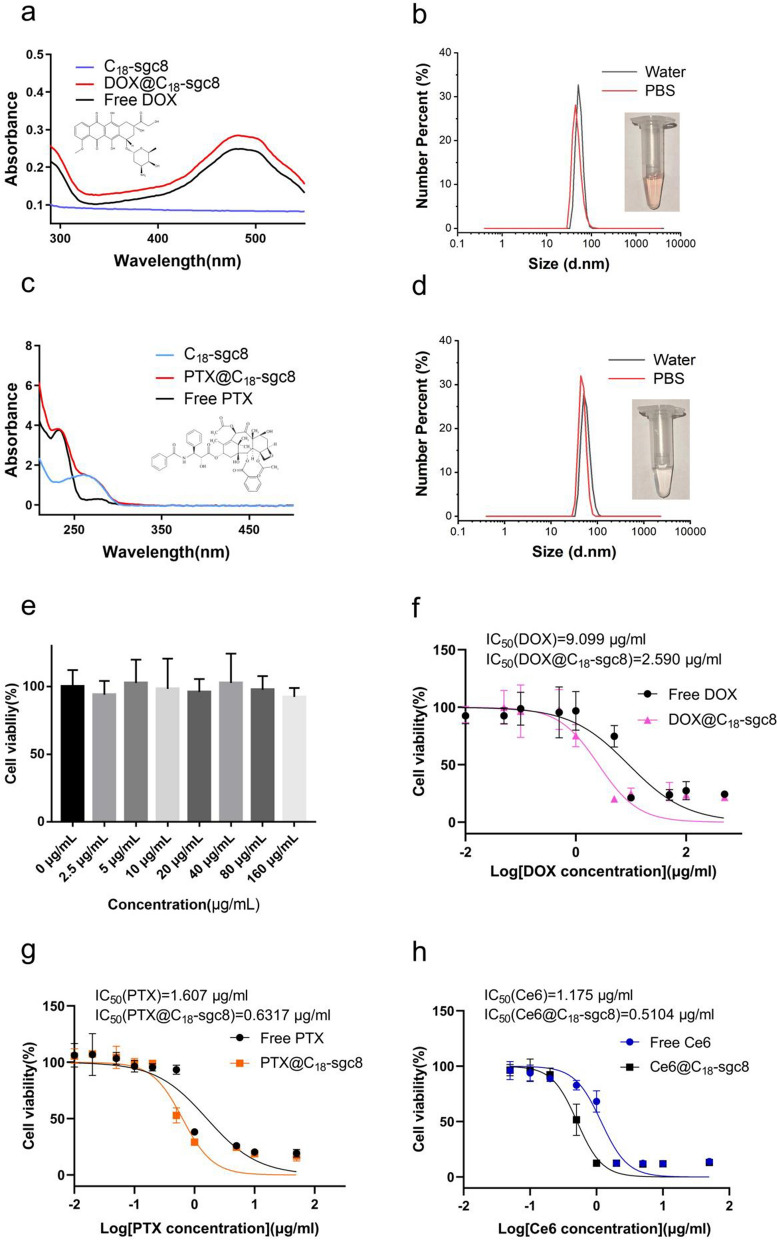
Validation of the versatility of C_18_-sgc8 micelles. **a** UV*–*vis spectra of DOX, C_18_-sgc8 and DOX@C_18_-sgc8. **b** DLS measurements of DOX@C_18_-sgc8 micelles in water (black) and PBS (red). **c** UV*–*vis spectra of PTX, C_18_-sgc8 and PTX@C_18_-sgc8. **d** DLS measurements of PTX@C_18_-sgc8 micelles in water (black) and PBS (red). **e** Toxicity of C_18_-sgc8 at different concentrations on HeLa cells, as measured by CCK-8 assay. **f** Efficient killing of HeLa cells after treatment with different concentrations of DOX@C_18_-sgc8 or DOX. **g** Efficient killing of HeLa cells after treatment with different concentrations of PTX@C_18_-sgc8 or PTX. **h** Efficient killing of HeLa cells after treatment with different concentrations of Ce6@C_18_-sgc8 or Ce6 exposure to 660 nm light. Data are expressed as mean ± standard deviation (n = 3)

Previous studies have experimentally demonstrated that C_18_-sgc8 loaded with the hydrophobic photosensitizer Ce6 is efficacious in photodynamic therapy. It follows that C_18_-sgc8 could be used as a versatile nucleic acid micelle nanoplatform for loading other drugs. To explore this idea, doxorubicin (Brand name: adriamycin) and paclitaxel were chosen to synthesize DOX@C_18_-sgc8 and PTX@C_18_-sgc8, respectively, and their particle sizes were measured in aqueous solution or PBS. For DOX@C_18_-sgc8, the median particle size in water or PBS is about 51.75 nm and 50.75 nm, respectively (Fig. 4b), while the median particle size of PTX@C_18_-sgc8 in water or PBS is about 43.82 and 50.75 nm, respectively (Fig. 4d). The particle sizes of DOX@C_18_-sgc8 and PTX@C_18_-sgc8 were both significantly larger than the previously measured size of C_18_-sgc8, which can be attributed to the loading of these drugs into the hydrophobic cavity inside the micelle. In addition, fluorescence spectroscopy (Additional file 1: Fig. S3) and UV-V is absorption spectroscopy were performed to compare the absorption spectra of DOX@C_18_-sgc8 and PTX@C_18_-sgc8 with those of free DOX and PTX. Results showed that DOX@C_18_-sgc8 and free DOX share a similar characteristic absorption peak at 480 nm (Fig. 4a); meanwhile, PTX@C_18_-sgc8 and free PTX share a characteristic peak at 230.5 nm (Fig. 4c), both of which indicated that C_18_-sgc8 could be used to load drug molecules using the same synthetic method. As shown in Additional file 1: Fig. S4, the maximum drug loading efficiency of C_18_-sgc8 was 89.48% for Ce6 and 86.36% for PTX. These results confirm that C_18_-sgc8 is a promising platform for loading a variety of hydrophobic drugs intended for use in a range of applications beyond PDT.

To explore the potential of C_18_-sgc8 nanomicelles in biomedicine, experiments were performed to evaluate the effect of empty C_18_-sgc8 on HeLa cell viability using the CCK-8 method. First, the cell viability of empty C_18_-sgc8 micelles with concentrations ranging from 0 to 160 µg/mL was evaluated after incubation with HeLa cells for 24 h. Results showed no significant change in cell viability with increasing C_18_-sgc8 concentrations (Fig. 4e), indicating that C_18_-sgc8 has good biosafety and biocompatibility. The cell-killing potential of drug-loaded micelles was also evaluated by determining IC_50_ values (the concentration at which a substance exerts half of its maximal inhibitory effect) after incubating micelles with HeLa cells. After incubation at 4 °C for 2 h, the drug-loaded micelles were washed and replaced with fresh medium for another 24 h incubation, followed by performing CCK-8 assay to determine cell viability. DOX@C_18_-sgc8 (IC_50_ = 2.590 µg/mL) exhibited higher cytotoxicity compared to free DOX (IC_50_ = 9.099 µg/mL) at the same drug concentration (Fig. 4f). Meanwhile, PTX@C_18_-sgc8 (IC_50_ = 0.6317 µg/mL) was more cytotoxic than free PTX (IC_50_ = 1.607 µg/mL) at the same drug concentration (Fig. 4g). In addition, as shown in Fig. 4h, the cytotoxicity of Ce6@C_18_-sgc8 (IC_50_ = 0.5104 µg/mL) was found to be higher than that of free Ce6 (IC_50_ = 1.175 µg/mL) at the same laser irradiation (1 W/cm^2^ for 10 min) and drug concentration. This can likely be attributed to the targeted binding of aptamer-modified nucleic acid micelles, which readily enter cells via endocytosis, facilitating drug internalization. Compared to free drugs, micelles more efficiently diffuse into cells in larger quantities within a short period of time, resulting in a more potent killing effect on tumor cells. Based on the above results, C_18_-sgc8 nanomicelles can be used as an all-purpose nucleic acid micelle nanoplatform for loading various drugs. Moreover, these drug-loaded micelles exhibit enhanced cytotoxicity compared to free drugs, making them a promising approach for developing effective cancer therapy strategies.

**Fig. 5:**
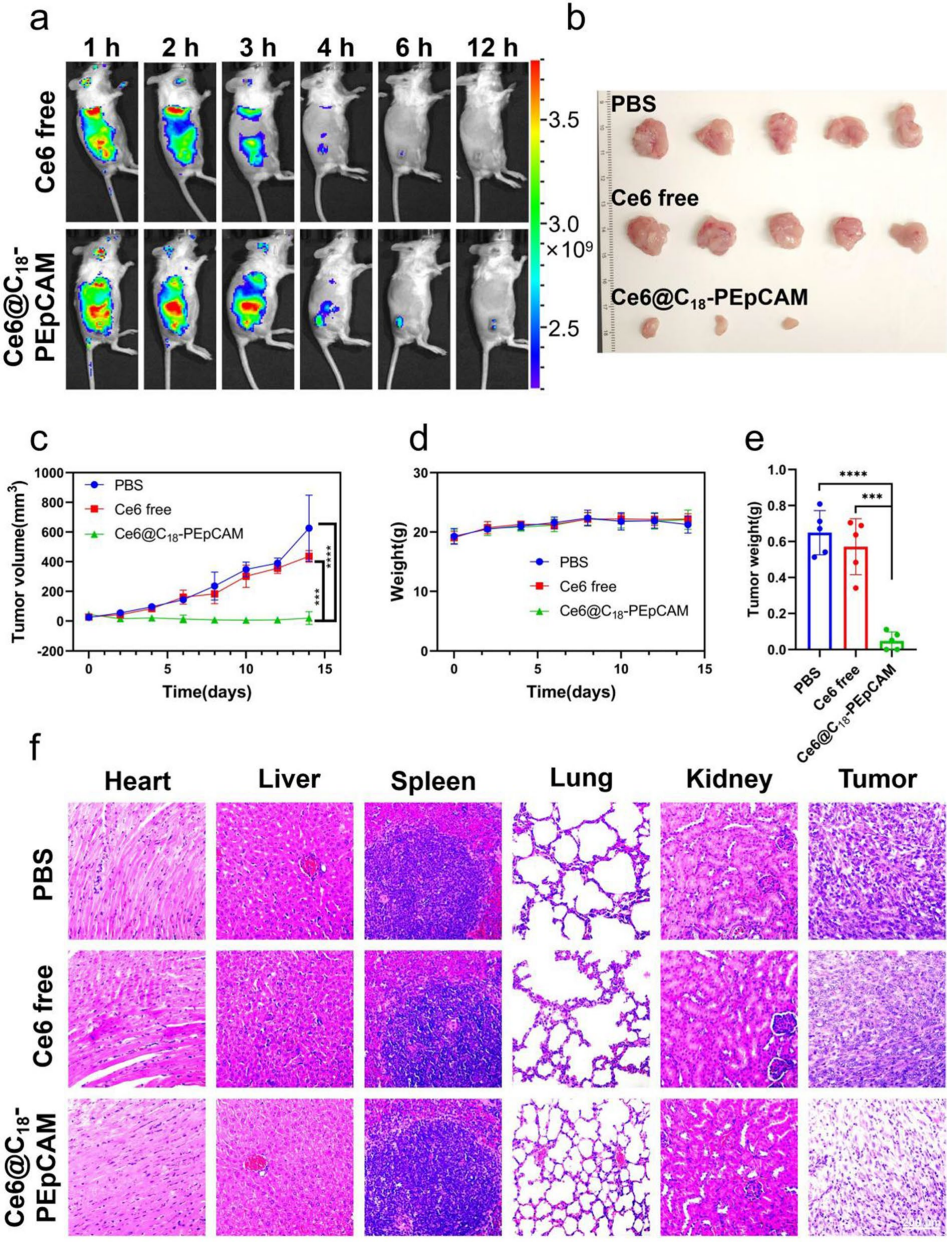
In vivo Fluorescence imaging and PDT treatment of 4T1 tumor-bearing mice with Ce6@C_18_-PEpCAM. **a** In vivo Fluorescence imaging of mice treated with free Ce6 or Ce6@C_18_-PEpCAM at different time points. **b** Photos of tumors collected from mice after various treatments on day 14. **c** Tumor growth curves of different treatment groups. **d** Body weight of 4T1 tumor-bearing mice after various treatments. **e** The tumor weight of mice after various treatments on day 14. ***p < 0.001 and ****p < 0.0001 were calculated by a student’s t-test (n = 5). **f** Hematoxylin and eosin (H&E) staining results of major organs and tumors of mice after various treatments

In light of the remarkable in vitro targeted photodynamic therapy effects demonstrated by aptamer-based self-assembled nanomicelles, our research efforts extended to investigating the in vivo targeted photodynamic therapy effects of these nanomicelles. As a proof of concept, we chose to synthesize aptamer-based self-assembled nanomicelles using a nucleic acid aptamer with specific affinity for epithelial adhesion molecules known to be highly expressed in 4T1 cells. To optimize cost-effective, we modified a portion of polyvalent hydrophobic poly(maleic anhydride-alt-1-octadecene) by coupling it with poly(ethylene glycol) and subsequently conjugated it with the EpCAM aptamer to obtain a block copolymer named C_18_-PEpCAM. This block copolymer was then used in the self-assembly process to load Ce6, resulting in the formulation of Ce6@C_18_-PEpCAM. To validate the tumor-targeting efficacy of the aptamer-based micelles, Balb/c mice bearing 4T1 tumors were administered either free Ce6 or Ce6@C_18_-PEpCAM, and their biodistribution was assessed using in vivo fluorescence imaging. As shown in Fig. 5a, Ce6 fluorescence intensity at the tumor site was significantly higher in the Ce6@C_18_-PEpCAM group compared to the free Ce6 group at the corresponding site. In addition, we harvested organs of interest for fluorescence imaging at 6 h after injection, as shown in Additional file 1: Fig. S5. Mice injected with Ce6@C_18_-PEpCAM showed significantly increased Ce6 accumulation within the tumors compared to mice receiving free Ce6. This indicated increased Ce6 accumulation in tumors with Ce6@C_18_-PEpCAM treatment. To investigate the potential anti-tumor effect, mice were divided into treatment groups (PBS, free Ce6, and Ce6@C_18_-PEpCAM) and irradiated with 100 mW/cm^2^ of 660 nm near-infrared light for 10 min after 6 h post-injection on days 0, 3, and 6, respectively. Tumor growth was monitored and after 14 days the tumors were excised and weighed. The Ce6@C_18_-PEpCAM group showed a significant delay in tumor growth compared to the control group (Fig. 5b, c, e and Additional file 1: Fig. S6). Compared to other groups, histological analysis of tumor tissues confirmed extensive destruction and necrosis of tumor cells in the Ce6@C_18_-PEpCAM group (Fig. 5f). During the 14 days, there were no abnormal changes in body weight or fatalities observed in any group, indicating the biocompatibility of Ce6@C_18_-PEpCAM (Fig. 5d). Histological analysis of major organs, including heart, liver, spleen, lung and kidney, further confirmed the biocompatibility of Ce6@C_18_-PEpCAM (Fig. 5f).

## Conclusions

In summary, a C_18_-sgc8 nanometer micelle system able to self-assemble into spheres in water has been successfully established. This unique structure, composed of hydrophobic and hydrophilic chains, provides excellent stability and targeting ability for drug delivery. Compared to traditional aptamer micelles, this C_18_-sgc8 micelle does not require crosslinking and is able to maintain its structural integrity during cellular interactions, enhancing its ability to deliver drugs to target cells. By loading hydrophobic drugs into the micelle complex, the versatility of C_18_-sgc8 as an all-purpose nucleic acid micellar nanoplatform has been established. Overall, results suggest that C_18_-sgc8 may be a promising approach for targeted delivery of hydrophobic drugs for biomedical applications.

### Supplementary Information

**Additional file 1:** **Figure S1**. Particle size and PDI of C_18_-sgc8 in 7 weeks. **Figure S2**. CMC values of C_18_-sgc8. **Figure S3**. Fluorescence spectra of DOX@C_18_-sgc8. **Figure S4**. Drug loading efficiency of C_18_-sgc8. **Figure S5**. Ex vivo Fluorescence imaging of Ce6@C_18_-PEpCAM. **Figure S6**. Photos of mice bearing 4T1 tumor.

## Data Availability

All data generated or analysed during this study are included in this published article.
