# Supplementary material for: Aptamer-based self-assembled nanomicelle enables efficient and targeted drug delivery
Source: J Nanobiotechnology. 2023 Nov 9;21:415. doi: 10.1186/s12951-023-02164-y (PMC10634091; doi:10.1186/s12951-023-02164-y)
Supplement: Supplementary file 1 — Additional file 1: Figure S1. Particle size and PDI of C18-sgc8 in 7 weeks. Figure S2. CMC values of C18-sgc8. Figure S3. Fluorescence spectra of DOX@C18-sgc8. Figure S4. Drug loading efficiency of C18-sgc8. Figure S5. Ex vivo Fluorescence imaging of Ce6@C18-PEpCAM. Figure S6. Photos of mice bearing 4T1 tumor. [file 12951_2023_2164_MOESM1_ESM.docx]

**Additional file 1**

Aptamer-based self-assembled nanomicelle enables efficient and targeted drug delivery

Ganghui Chen^a,b^, Dongsheng Mao^a^, Xuan Wang^a^ , Jingqi Chen^a^ , Chao Gu^a^ , Shuqin Huang^b^, Yu Yang^a*^, Fang Zhang^b*^, Weihong Tan^a^

^a^ Institute of Molecular Medicine (IMM), Renji Hospital, Shanghai Jiao Tong University School of Medicine, and College of Chemistry and Chemical Engineering, Shanghai Jiao Tong University, Shanghai 200240, China.

^b^ College of Biological Science and Engineering, Fuzhou University, Fuzhou 350108, People’s Republic of China.

**Materials**

Oligonucleotides were synthesized by Beijing Hippocampus Jatronic Biological Co., Ltd. and purified by high-performance liquid chromatography (HPLC). C18PMH, methanol, Ce6, DOX, PTX, EDC, and DMSO were purchased from Sigma-Aldrich, USA. Paraformaldehyde and agarose powder were purchased from Shanghai Aladdin Biochemical Technology Co. DNA marker was purchased from BBI Life Sciences Corp. The HeLa cell line (human cervical cancer cells) and Ramos cell line (human lymphoma cells) were obtained from the Cell Bank of Chinese Academy of Science (Shanghai, China). Hoechst-33342, SOSG fluorescent probe and DCFH-DA were purchased from Beyotime Biotechnology. Annexin V-FITC/PI, CCK-8, PBS, FBS, DMEM medium and 1640 medium were purchased from Thermo Fisher Scientific.

**Methods**

**Preparation of C_18_-sgc8**

An amphiphilic polymeric material was synthesized using an amino-modified nucleic acid aptamer (NH_2_-sgc8) with poly(maleic anhydride-alt-1-octadecene) (C_18_PMH). Specifically, C_18_PMH (1 mg) was first activated by adding 1.14 mg EDC and 0.66 mg NHS in 1 ml dichloromethane (DCM) at room temperature for 0.5 h. Then， 0.1 ml of activated C_18_PMH was taken and added to 0.1 ml of 5.7 mM NH_2_-sgc8, followed by 2 µl of triethylamine. The mixture was stirred vigorously for 24 h. The temperature was adjusted to 30℃ to allow the DCM solvent to evaporate. The residue was dissolved in water and dialyzed in a dialysis bag (MWCO = 14 kDa) at room temperature for 2 days, followed by storing the product at 4℃.

**Preparation of C18-PEpCAM**

Firstly, C18PMH-PEG-NH2 was synthesised according to our previously developed protocol. Briefly, 10 mg (1 aliquot) of C18PMH, 143 mg (1 aliquot) of mPEG-NH2(5k), and 50 mg (0.5 aliquot) of NH2mPEG(5k)-BOC (Polymere, Germany) were mixed in dichloromethane under stirring to form a homogeneous solution. EDC (2 aliquots) and TEA (8 aliquots) were then added under magnetic stirring. After stirring for 24 hours at room temperature, the solution was dialysed in a dialysis bag (MWCO = 14 kDa) for 2 days to remove unreacted PEG polymer and other reagents. Mix by adding COOH-EpCAM at 10:1 according to NH2mPEG(5k)-BOC. After 24 hours of reaction at room temperature, C_18_PMH-PEG-EpCAM (termed C_18_-PEpCAM) is purified by aqueous dialysis using a 14 kDa membrane and then lyophilised.

**Morphology observation of C_18_-sgc8 micelles**

The morphology of nanoparticles was observed by transmission electron microscopy (TEM). Aqueous solutions of C_18_-sgc8 were taken and diluted 10 and 50 times, respectively, and the samples were dropped onto a copper grid to form a hemispherical liquid surface. After they were allowed to dry naturally, 3% phosphotungstic acid was added dropwise for staining. The staining solution was then blotted with filter paper around the copper mesh, and ultrapure water was added dropwise on the copper mesh and blotted with filter paper. After drying at room temperature, the samples were sent to Zhenjiang Specialized Testing Company in Suzhou for TEM and TEM EDS mapping observations.

**C_18_-sgc8 micelle-loaded drugs**

Ce6 (1 mg), PTX (1 mg) and DOX (1 mg) powders were dissolved in 1 ml of methanol, and then 0.2 ml were added to 1 mg of C_18_-sgc8, stirred and sonicated, followed by stirring at room temperature overnight. The synthetic micelle drug solution was purified by centrifugation at 14800 rpm for 5 minutes to collect the supernatant and remove the precipitated free drug, followed by storing the product at 4°C.

**Stability of C_18_-sgc8 micelles**

The stability of C_18_-sgc8 micelles was evaluated by measuring the change in particle size at different time. Synthesized C_18_-sgc8 was added to PBS or water, respectively, and then placed at 25℃. Stability of the C_18_-sgc8 micelles was determined by periodically measuring and statistically analyzing the changes in particle size and PDI values of the C_18_-sgc8 micelles in three repetitions.

**Characterization by agarose gel electrophoresis**

First, 4 g of agarose powder and 100 mL of 1×TAE buffer were added sequentially to a beaker and then boiled until completely melted. The prepared gel was quickly poured into an agarose gel-making rack and left at room temperature for 30 minutes until it cooled and solidified into a block. Four percent of the prepared agarose gel was added to 1× TAE buffer (pH=8.0), and then 2 μL of 6×loading buffer containing GelRed were mixed with 10 μL of sample and added sequentially to each lane of the agarose gel block in turn for 30 minutes at 120V. After electrophoresis, the GelRed channel was selected for imaging under the fluorescence imaging system.

**Fluorescence spectroscopy determination**

The free photosensitizers Ce6 and adriamycin (DOX), as well as Ce6@C_18_-sgc8 and DOX@C_18_-sgc8, were characterized by fluorescence spectroscopy, respectively. The excitation wavelength of Ce6 was set to 480 nm, the emission wavelength was set to 500-700 nm, and the incident slit and exit slit were set to 5 nm and 10 nm, respectively. The excitation wavelength of DOX was set to 365 nm, the emission wavelength was set to 500-700 nm, and the incident slit and exit slit were set to 5 nm and 10 nm, respectively, for fluorescence spectroscopy.

**Determination of critical micelle concentration (CMC)**

The critical micelle concentration of C_18_-aptamer was determined by light scattering using the method described in the literature[1, 2]. First, after passing C18-sgc8 through a 0.22 μm filter membrane, aqueous solutions of C18-sgc8 at different concentrations (10^-8^ to 10^-1^ mg/ml) were configured, and the intensity of scattered light was measured using a Malvern & Nano ZS to make two fitted lines at the maximum turning point and calculate the value at the intersection.

**Confocal microscopy for fluorescence imaging to verify specific binding**

We assessed the specific binding capacity of C_18_-sgc8 by laser confocal microscopy. First, 3×10^4^ HeLa and Ramos cells were inoculated separately on confocal-specific culture dishes and incubated in a cell incubator containing 5% CO_2_ for 4 h at 37°C. Then, 500 μL of fresh culture medium containing 200 nM C_18_-sgc8-FITC were added and incubated for 1 h at 37°C. Free C_18_-sgc8-FITC was then gently washed with DPBS, followed by staining with Hoechst 33342 (5 μg/ml) for 10 min and washing gently with DPBS 3 times to remove dye not bound to the nuclei. Finally, fluorescent pictures were taken under a laser confocal microscope providing Hoechst excitation at 405 nm and measured from 430 nm to 480 nm, and the FITC excitation at 488 nm and measured from 500 nm to 550 nm.

**Flow cytometry analysis of micellar targeting**

Flow cytometry was used to validate the ability of C_18_-sgc8 to specifically bind tumor cells. First, an amphiphilic polymeric material was synthesized using an amino-modified nucleic acid aptamer (NH_2_-sgc8) with poly(maleic anhydride-alt-1-octadecene) (C_18_PMH), and the specific binding capacity of C_18_-sgc8 was assessed using Lipid-sgc8-FITC and Cholesterolsgc8-FITC as controls. Specifically, wells of a 24-well plate were inoculated with 5. 0×10^4^ HeLa cells and Ramos cells and incubated for 12 h before adding 200 μL of Lipid-sgc8-FITC, C_18_-sgc8-FITC and Cholesterol-sgc8-FITC at a concentration of 200 nmol, respectively. The mixtures were co-cultured for 1 h at 4°C protected from light. Afterwards, cells were gently washed with binding buffer, followed by centrifugation at 1000 rpm. Cells were collected, washed three times and collected again for flow cytometric analysis in the FITC channel. The obtained data were analyzed and plotted using FlowJo v 10.

**Cell endocytosis experiment**

First, 3×10^4^ HeLa cells were seeded into 35 mm optical confocal culture dishes and incubated overnight with Ce6@C_18_-sgc8 (containing 5.0 mg/L Ce6) and equivalent concentrations of Ce6 in a cell culture incubator containing 5% CO2 at 37°C for 0, 0.5, 1, 2 and 4 h. Cells were gently rinsed with DPBS 3 times and then incubated with Hoechst 33342 (5 μg/ml) for 10 minutes. Cells were then washed gently with DPBS 3 times, rinsed and fixed with 4% paraformaldehyde for 10 min. Finally, the cellular uptake of the different materials was examined by Lycra confocal laser scanning microscopy with Hoechst excitation selected at 405 nm and measured from 430 nm to 480 nm and Ce6 fluorescence excitation selected at 488 nm and measured from 600 nm to 700 nm.

**Evaluation of SOG Ability of Ce6 and Ce6@C_18_-sgc8**

Singlet oxygen was measured by using SOSG (Molecular Probes). PBS, free Ce6, and Ce6@C_18_-sgc8 (Ce6 concentration consistent with Free Ce6) were first irradiated under laser light (660 nm, 1 W/cm^2^) for different times (0, 2, 4, 6, 8, 10 min), respectively. SOSG (2 μM) was then added, and fluorescence intensity at 525 nm was detected at an excitation wavelength of 494 nm.

### Generation of Intracellular ROS

### First, 5x10^4^ HeLa cells were inoculated in 35 mm confocal dishes and incubated for 12 hours. The medium was then washed off, and 500 μL of serum-free medium containing Ce6 or Ce6@C18-sgc8 were added. After 2 h of incubation, the medium was replaced with fresh medium, and 10 μL DCFH-DA (0.03 mg/mL) were added to the medium and incubated for a further 30 min, followed by irradiation with a 660 nm laser (1 W/cm^2^) for 10 min. Finally, cells were washed with DPBS and imaged using confocal microscopy.

**Biocompatibility of C_18_-sgc8**

The biocompatibility of cells was measured with empty C18-sgc8 micelles using the CCK-8 method. HeLa cells were inoculated in wells of a 96-well plate at a density of 1×10^4^ cells per well and a volume of 100 μL of medium per well. After incubation in a cell incubator at 37°C with 5% CO2 for 12 h, C_18_-sgc8 was added to wells at concentrations of 0, 2.5, 5, 10, 20, 40, 80, and 160 μg/ml, respectively, with three replicate wells set for each concentration. After co-incubation for 24 h, cells were washed 3 times with DPBS, and 90 μL of fresh DMEM medium were added to each well. Next, 10 μL of CCK-8 were added to each well and incubated for 1 h in a cell incubator with 5% CO2 at 37°C. Finally, absorbance values at 450 nm were measured and cell viability was calculated as

Cell Viability (%) = (A _experimental group_ - A _blank_)/(A _control group_ - A _blank_)×100%

**C_18_-sgc8-loaded drug killing effect on HeLa cells**

The cytotoxicity of Ce6@C_18_-sgc8, PTX@C_18_-sgc8 and dox@C_18_-sgc8 was evaluated by the CCK-8 assay. HeLa cells were first inoculated *in vitro* at a density of 1×10^4^ cells per well in a 96-well plate with a volume of 100 μL of medium per well and incubated in a cell incubator with 5% CO2 at 37°C for 12 h. After incubation, different concentrations of Ce6@C_18_-sgc8, PTX@C_18_-sgc8 and DOX@C_18_-sgc8 were added to the corresponding wells. Three replicate wells were set up for each concentration. After 2 hours, cells were washed 2-3 times with DPBS, respectively, and 100 μL of fresh DMEM medium were added to each well. In addition to Ce6@C_18_-sgc8 cell killing, the cells were also subjected to laser light irradiation using a 660 nm laser with a power of 1 W/cm2 for 10 min. Cells were then incubated in a cell incubator at 37°C with 5% CO2 for 24 h. 10 μL of CCK-8 were then added to each well and incubated in a cell incubator at 37°C with 5% CO2 for 1 h. Finally, absorbance values at 450 nm were measured, and cell viability was calculated as

Cell Viability (%) = (A _experimental group_ - A _blank_)/(A _control group_ - A _blank_)×100%

**In vivo and in vitro fluorescence imaging**

4T1 cells were resuspended in PBS and subcutaneously injected into the right side of female BALB/C nude mice (4 weeks)， 3×10^6^ cells/site to establish tumor bearing mice. When the tumor size reached~200 mm3, the biological distribution and imaging were studied.

The free CE6 and Ce6@C18-PEpCAM (the dose of CE6 10 mg/kg of the total body weight of mice) were intravenously injected into tumor bearing mice. The fluorescence images (excitation, 680nm; emission, 710nm) at the required time intervals (1, 2, 3, 4, 6, and 12 hours) were collected on the fluorescence imaging system. Fluorescence images of resected tumors and organs are also obtained on the imaging system with the same parameters as previously mentioned.

**animal model**

Balb/c mice aged 6 weeks were selected and placed in the research animal facility according to the animal protocol approved by us. All mouse experiments were carried out in accordance with the standards specified by the national administration. The mice were divided into three groups, and 5×10^6^ 4T1 cancer cells were subcutaneously injected into the right leg. Treatment was started when the tumor size was 100 mm^3^. Intravenous injections of PBS, free Ce6 or Ce6@C_18_-PEpCAM were performed at days 0, 3 and 6, respectively. After 6 hours of intravenous injection, the tumor site was irradiated with 660 nm laser for 10 minutes (1W/cm^2^). The tumor size was measured every 2 days. The length and width of the tumor were measured by vernier caliper, and the growth of the tumor was evaluated.

**Histological examination**

After 2 weeks of treatment, the heart, liver, spleen, lung, kidney, tumor and other organs of mice were collected and fixed in 4% paraformaldehyde at 4℃ overnight. Then the tissue specimens were embedded in paraffin, cut and stained with H&E. Finally, the main organ sections stained with h&e were visualized.

Table S1. Oligonucleotide Sequences in the Experiments

| Oligonucleotide | Sequence (5’-------3’) |
| --- | --- |
| Sgc8 | ATCTAACTGCTGCGCCGCCGGGAAAATACTGTACGGTTAGA |
| NH_2_-sgc8 | NH_2_-ATCTAACTGCTGCGCCGCCGGGAAAATACTGTACGGTTAGA |
| NH_2_-sgc8-FITC | NH_2_-ATCTAACTGCTGCGCCGCCGGGAAAATACTGTACGGTTAGA-FITC |
| Lipid-sgc8-FITC | Lipid-ATCTAACTGCTGCGCCGCCGGGAAAATACTGTACGGTTAGA-FITC |
| Cho-sgc8-FITC | Cho-ATCTAACTGCTGCGCCGCCGGGAAAATACTGTACGGTTAGA-FITC |
| COOH-EpCAM | COOH-CACTACAGAGGTTGCGTCTGTCCCACGTTGTCATGGGGGGTTGGCC |


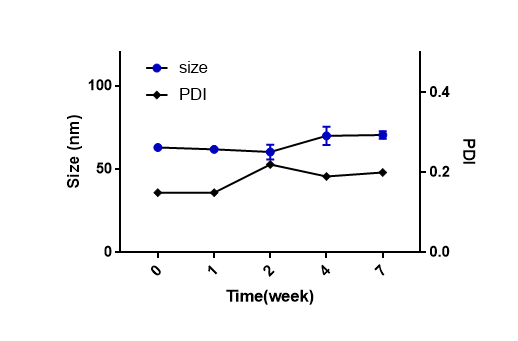


**Figure S1**. Particle size and PDI of C_18_-sgc8 in water at different time points over a 7-week period, as determined by Malvern & Nano ZS for C_18_-sgc8 stored at 4°C for extended periods of time.


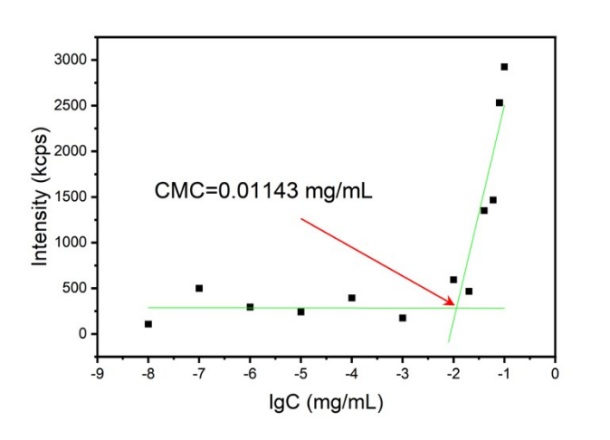


**Figure S2**. CMC values of C18-sgc8 in aqueous solution were determined using the light scattering method to determine the critical micelle concentration of C18-aptamer.


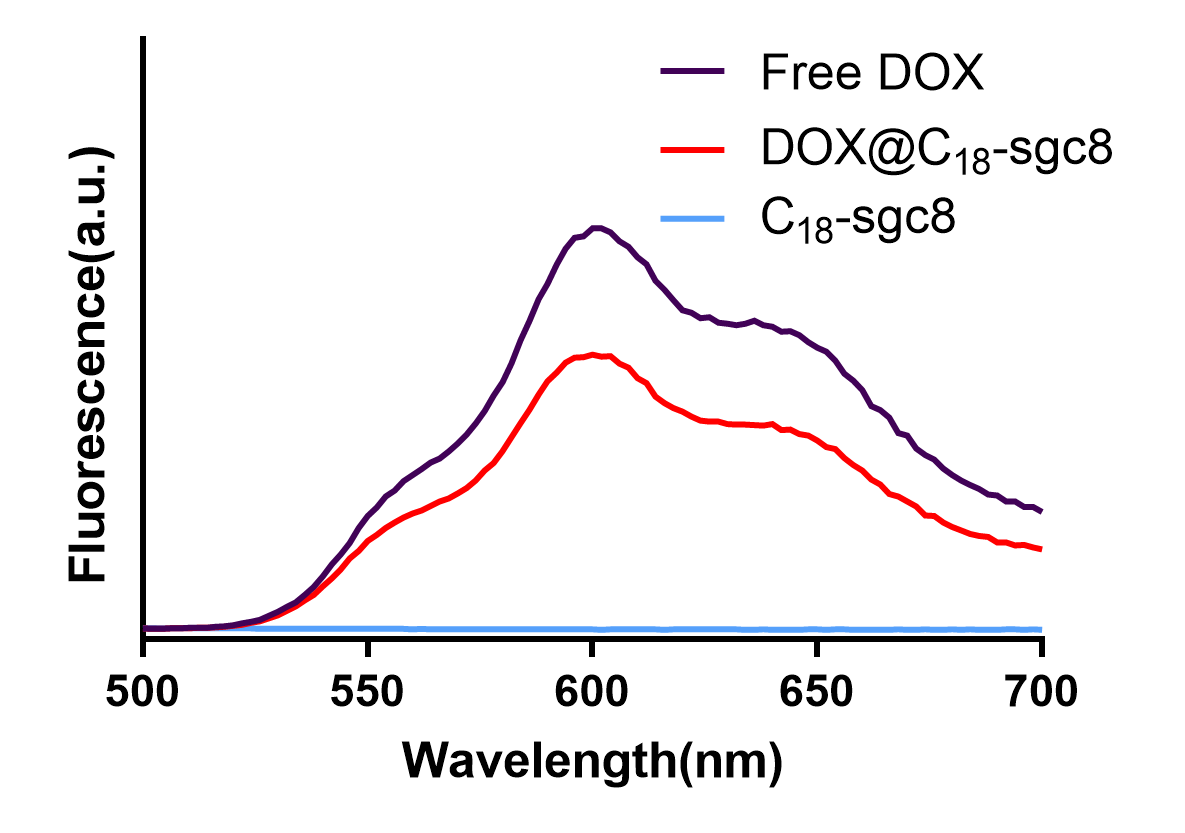


**Figure S3**. Excitation wavelength at 365 nm is used to detect the fluorescence spectrum of Free DOX, DOX@C_18_-sgc8 and C_18_-sgc8 set to a known absorbance between 500 and 700 nm.


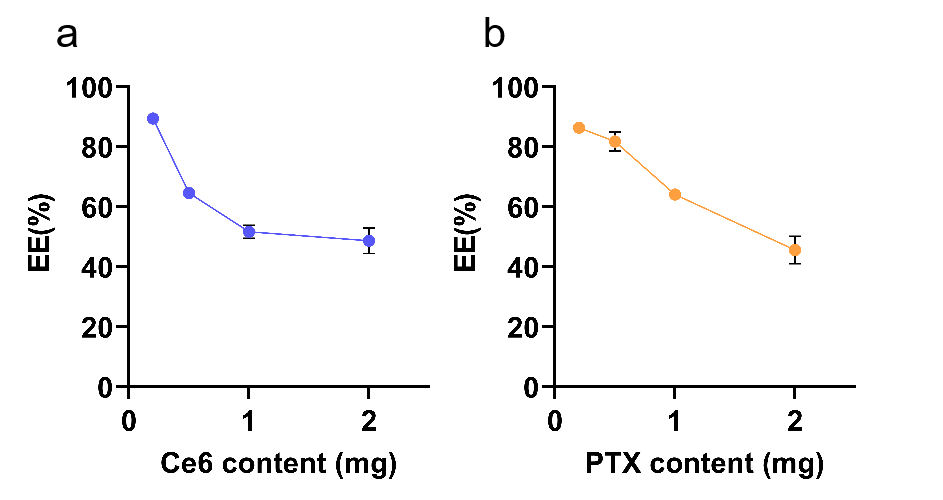


**Figure S4.** Drug loading efficiency of C18-sgc8 on hydrophobic drugs (a) Ce6 (b) PTX


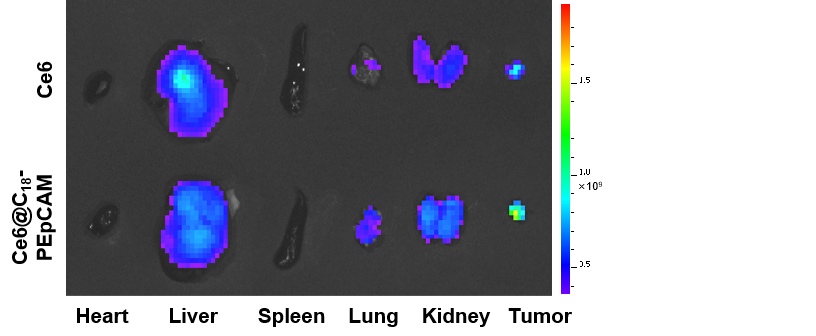


**Figure S5.** Ex vivo Fluorescence imaging of mice treated with free Ce6 or Ce6@C18-PEpCAM.


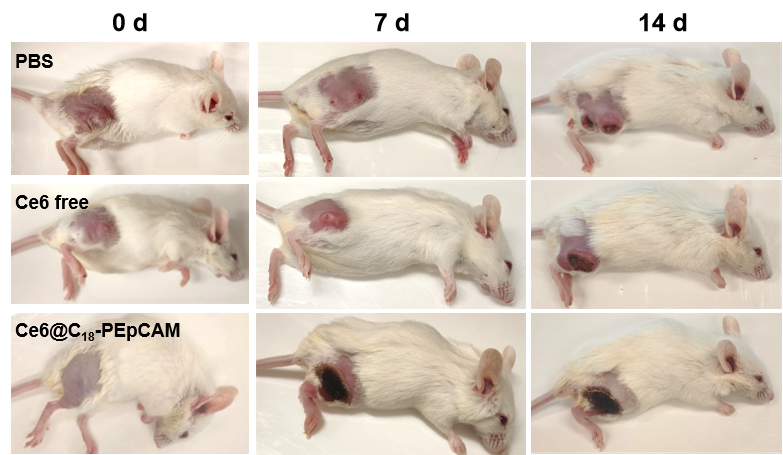


**Figure S6.** Photos of mice bearing 4T1 tumor on day 0, 7 and 14 after different treatments.

**References**

1. Horiuchi S, Winter G. CMC determination of nonionic surfactants in protein formulations using ultrasonic resonance technology. Eur J Pharm Biopharm. 2015;92:8-14.

2. Topel Ö, Çakır BA, Budama L, Hoda N. Determination of critical micelle concentration of polybutadiene-block-poly(ethyleneoxide) diblock copolymer by fluorescence spectroscopy and dynamic light scattering. J Mol Liq. 2013;177:40-43.
